# Synergistic antitumor effects of combined deguelin and cisplatin treatment in gastric cancer cells

**DOI:** 10.3892/ol.2014.2368

**Published:** 2014-07-22

**Authors:** ZHENGGUANG LI, CHANGPING WU, JUN WU, MEI JI, LIANGRONG SHI, JINGTING JIANG, BIN XU, JINJIN YUAN

**Affiliations:** Department of Oncology, The Third Affiliated Hospital of Soochow University, Changzhou, Jiangsu 213003, P.R. China

**Keywords:** gastric cancer, deguelin, cisplatin, synergism

## Abstract

Deguelin is a naturally occurring rotenoid with marked chemopreventive and antitumor activity. Cisplatin, characterized by damaging DNA, is widely used in the chemotherapy of malignancies, including gastric cancer. The present study investigated whether synergistic effects exist between the combination of deguelin and cisplatin, and the possible mechanism *in vitro*. The interactive effects of deguelin in combination with various concentrations of cisplatin were evaluated in the human gastric cancer MGC-803 cell line. The inhibition of cell proliferation was determined by 3-(4,5)-dimethylthiazol(-2-yl)-2,5-diphenyltetrazolium bromide assay and the mechanisms underlying the effects were further evaluated by western blot analysis. The results revealed that the combined treatment of deguelin and cisplatin exhibited a marked inhibition of MGC-803 cell proliferation when compared with that of the single therapies *in vitro*. In addition, isobologram analysis revealed that this combined treatment showed a synergistic effect. These observations may have promising therapeutic value for gastric cancer and thus warrant further investigation.

## Introduction

Gastric cancer is the fourth and fifth most prevalent cancer diagnosed in males and females, respectively, worldwide and the third and fifth most common cause of cancer-related mortality in males and females, respectively (1). Surgical resection remains the mainstay of potentially curative treatment. However, local-regional and distant recurrences are common, and the treatment outcome is far from satisfactory (2). Thus, surgery alone is insufficient for the treatment of the majority of patients. In addition, gastric cancer is often diagnosed at an advanced stage, which is unresectable. Therefore, chemotherapy and chemoradiation are important for the treatment of gastric cancer, particularly in patients with advanced and metastatic disease (3,4).

Platinum-based drugs have been widely used and extensively studied in anticancer therapy based on their ability to covalently bind to DNA (5,6). At present, cisplatin remains the most frequently used chemotherapy for different malignances. In gastric cancer, regimens that contain platinum-based drugs have been successfully administered in perioperative and postoperative chemotherapy, as well as in chemotherapy for advanced/metastatic disease, with significant efficacy (7–10). However, despite the efficacy of platinum-based drugs against gastric cancer, concerns exist regarding the use of these agents. One concern is that cancer cells exhibit an inherent or acquired refractory to platinum, which reduces its efficacy resulting in disease relapse (11,12). An additional concern is their potent toxicity and side effects, which significantly limit the tolerable therapy dose.

**Figure 1:**
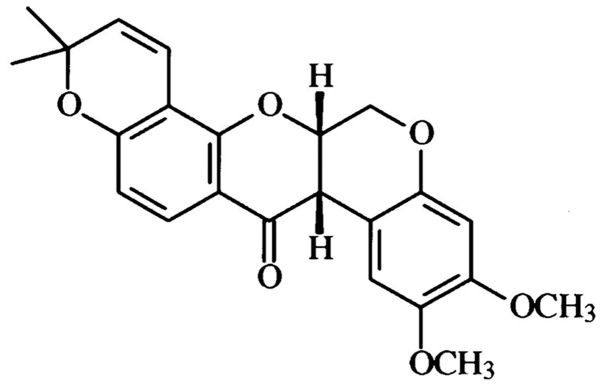
Chemical structure of deguelin.

Deguelin is a natural rotenoid isolated from several plant species, including *Mundulea sericea* (Fig. 1). Deguelin has been found to exhibit marked chemopreventive and antitumor activity against cancer, in various model systems (13–15). Recently, it was reported that deguelin induces DNA damage by reducing the expression of DNA repair genes in human non-small cell lung cancer cells (16). The mechanism of platinum-refractory or platinum-resistance *in vivo* is multi-factorial, including increased tolerance to platinum-induced and enhanced repair of DNA damage (17–22). Thus, to investigate whether deguelin synergistically potentiates the antitumor effects of cisplatin, the interactive effects of deguelin and cisplatin *in vitro* were examined using the gastric carcinoma MGC-803 cell line. In addition, the synergistic effects and possible mechanisms were analyzed.

## Materials and methods

### Materials

3-(4,5)-dimethylthiazol(-2-yl)-2,5-diphenyltetrazolium bromide (MTT), dimethyl sulfoxide (DMSO), propidium iodide (PI) and cisplatin were purchased from Sigma-Aldrich (St. Louis, MO, USA). The total protein extraction and comet assay kits were purchased from Nanjing Keygen Biotech., Co., Ltd. (Nanjing, China). All the chemicals used in this study were analytically pure and of culture grade. The primary antibodies against BRCA1 (monoclonal rabbit anti-human), ERCC1 (polyclonal rabbit anti-human), XRCC1 (polyclonal rabbit anti-human) and GAPDH (monoclonal rabbit anti-human) were purchased from Cell Signaling Technology, Inc. (Beverly, MA, USA), and the Bio-Rad protein assay kit I (cat. no. 500–0001) was purchased from Bio-Rad (Hercules, CA, USA).

Deguelin was purchased from Sigma-Aldrich, dissolved in DMSO as a stock solution and stored at 4°C. The stock solution was then diluted in cell culture medium to a final concentration of 0.05% DMSO (V/V).

### Cell culture

The human gastric cancer MGC-803 cell line was purchased from the Type Culture Collection of the Chinese Academy of Sciences (Shanghai, China). The cells were cultured in RPMI-1640 medium (Life Technologies, Bedford, MA, USA) containing 10% heat-inactivated fetal bovine serum, 100 U/ml penicillin and 100 U/ml streptomycin (all Life Technologies, Bedford, MA, USA) at 37°C in a humidified atmosphere with 5% CO_2_.

### Cell viability assay

The cell viability of the treated cancer cells was determined using the MTT assay. Briefly, cells (4–5×10^3^) were seeded in 96-well plates and cultured for 24 h, followed by deguelin and cisplatin treatment. A volume of 10 μl of MTT (10 mg/ml) was added to each well and the cells were incubated for an additional 4 h at 37°C. The supernatant fluid was then removed and DMSO (150 μl/well) was added for 15–20 min. The optical densities were measured at a wavelength of 570 nm using the SpectraMAX M5 microplate spectrophotometer (Molecular Devices, LLC, Sunnyvale, CA, USA). All experiments were performed in triplicate. The effect of deguelin on the cell proliferation was presented as the cell growth inhibition, using the following formula: Inhibition rate (%) = (A_570_ of control − A_570_ of treated cells)/(A_570_ of control cells) × 100.

### Isobologram analysis

The interactions of the combination treatment of deguelin and cisplatin were analyzed by isobologram as previously described (23). The dose-dependent effects were determined for each compound and for one compound with fixed concentrations of the other. The combination index (CI) was calculated according to the following formula: CI = (*d*1/*Dx*1) + (*d*2/*Dx*2), where *Dx*1 is the concentration of drug 1 (deguelin) required to produce × percentage effect alone, and *d*1 is the concentration of drug 1 required to produce the same × percentage effect in combination with *d*2. Similarly, *Dx*2 is the concentration of drug 2 (cisplatin) required to produce × percentage effect alone, and *d*2 is the concentration of drug 2 required to produce the same × percentage effect in combination with *d*1. The CI values were defined as follows: <1, synergism; 1, additive; and >1, antagonism.

### Morphological analysis

Following culture and drug treatment as previously described, the morphological changes of the cells were observed. The cells were fixed in 70% ethanol following washing with phosphate-buffered saline (PBS). After examination for morphological changes with an inverted microscope (XDS-800C; Ahghai Caikon Optical Instrument Co., Ltd., Shanghai, China), the cells were stained with PI (1 μg/ml in PBS) and analyzed under a fluorescence microscope (Axiovert 200; Carl Zeiss, Göttingen, Germany).

### Western blot analysis

Following treatment with deguelin and cisplatin, 5×10^6^ cells were harvested and lysed in 1 ml lysis buffer (Nanjing Keygen Biotech., Co., Ltd.), and the protein concentration was determined using the Bio-Rad protein assay reagent (Bio-Rad). The samples were then denatured in sample buffer and the proteins were separated by sodium dodecyl sulfate-polyacrylamide gel electrophoresis. Next, the gels were electroblotted onto a polyvinylidene difluoride membrane, rinsed with Tris-buffered saline with Tween 20 [20 mM Tris, 500 mM NaCl and 0.1% Tween-20 (pH 7.6)] and blocked with 5% non-fat milk in blocking buffer. The membrane was incubated with the appropriate primary antibody overnight at 4°C. The membrane was then incubated with the appropriate peroxidase-conjugated secondary antibody and the immunoreactive bands were visualized using the enhanced chemiluminescence method.

### Statistical analysis

Data are presented as the mean ± standard deviation and statistical analyses were performed using the analysis of variance test. All data were analyzed using SPSS version 13.0 (SPSS, Chicago, IL, USA) and P<0.05 was considered to indicate a statistically significant difference.

## Results

### Effects of deguelin and cisplatin on cell proliferation

**Figure 2:**
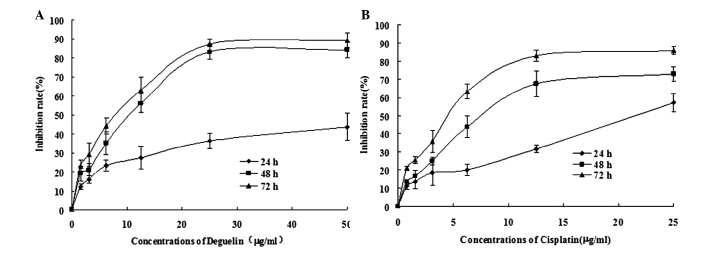
Inhibition of the proliferation of MGC-803 cells treated with (A) deguelin or (B) cisplatin. Cell viability experiments were performed following 24, 48 and 72 h of exposure to the compounds. Each point and vertical bar presents the mean ± standard deviation of three independent experiments.

The antiproliferative effects of deguelin and cisplatin alone on MGC-803 cells were investigated using the MTT assay. Deguelin and cisplatin treatment resulted in a dose- and time-dependent decrease in cell viability. Furthermore, when cells were treated with deguelin for 48 and 72 h, the IC_50_ values were 10.74 and 6.52 μg/ml, respectively. The IC_50_ values of cells treated with cisplatin for 24, 48 and 72 h were 22.90, 7.66 and 3.89 μg/ml, respectively (Fig. 2).

### Combined effects of deguelin and cisplatin

**Figure 3:**
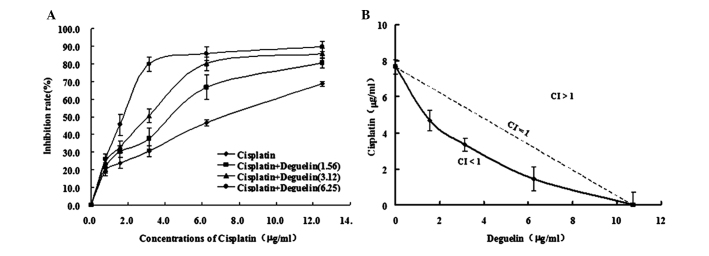
(A) Combined effects of deguelin and cisplatin and (B) isobologram analysis. MGC-803 cells were treated with cisplatin combined with deguelin at various concentrations (0, 1.56, 3.12 and 6.25 μg/ml). According to the data, when deguelin (1.56, 3.12 and 6.25 μg/ml) was combined with cisplatin (4.49, 3.34 and 1.45 μg/ml, respectively), the CI was <1 (0.72, 0.75 and 0.76, respectively). Each point and vertical bar presents the mean ± standard deviation of three independent experiments. CI, combination index.

The effects of cisplatin combined with deguelin in a series of concentrations were examined. When combined with deguelin, the IC_50_ of cisplatin in MGC-803 cells was found to decrease significantly, in a dose-dependent manner. For example, when the concentration of deguelin increased from 1.56 to 6.25 μg/ml, the IC_50_ of cisplatin was found to decrease from 4.69 to 1.45 μg/ml (Fig. 3).

To evaluate the interaction between deguelin and cisplatin, isobologram analysis was performed and the results showed that the CI was considerably <1 when deguelin and cisplatin were used in combination; CI=0.72 for 1.56 μg/mg deguelin combined with 4.49 μg/mg cisplatin; CI=0.75 for 3.12 μg/mg deguelin combined with 3.34 μg/mg cisplatin; and CI=0.76 for 6.25 μg/mg deguelin combined with 1.45 μg/mg cisplatin). These results indicated that deguelin and cisplatin exhibit synergistic effects which inhibit the growth of MGC-803 cells (Fig. 3).

**Figure 4:**
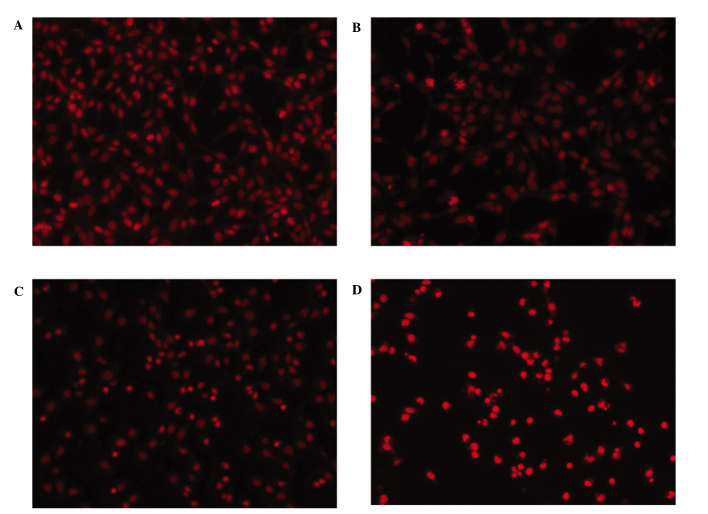
Morphological changes induced by deguelin and cisplatin. Fluorescence microscopy of propidium iodide-stained nuclei of the (A) untreated control group and MGC-803 cells treated with (B) 6.25 μg/ml deguelin, (C) 3 μg/ml cisplatin or (D) a combination of deguelin (6.25 μg/mg) and cisplatin (3 μg/mg) for 48 h (magnification, ×400). Apoptotic cells containing condensed and fragmented fluorescent nuclei were visible in the barbigerone-treated cells, but not in the untreated cells.

Furthermore, fluorescence microscopic examination of PI-stained cells was performed to confirm the synergistic effects between deguelin and cisplatin. Following treatment with 3 μg/ml cisplatin and 6.25 μg/ml deguelin, the cell number was significantly lower than that of the groups treated with cisplatin or deguelin alone. However, the number of apoptotic cells had increased (Fig. 4).

### Mechanistic studies of deguelin-cisplatin synergism

**Figure 5:**
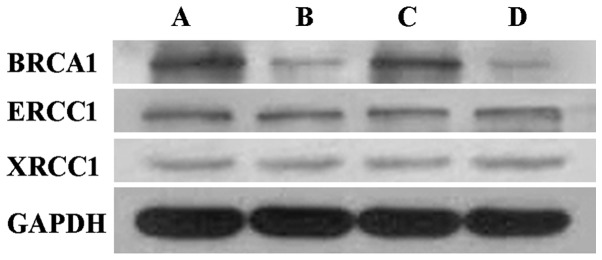
Effect of deguelin on the DNA damage repair genes. MGC-803 cells were treated with 6.25 μg/ml deguelin, 3 μg/ml cisplatin or the two drugs in combination (6.25 μg/mg deguelin and 3 μg/mg cisplatin) for 48 h and the expression levels of BRCA1, ERCC1 and XRCC1 were analyzed by western blot analysis. GAPDH was used as a loading control. BRCA1 was downregulated following exposure to deguelin, but ERCC1 and XRCC1 were not affected.

To identify the molecular mechanisms underlying the synergism between deguelin and cisplatin, the expression of various DNA damage repair genes was examined. The results revealed that BRCA1 expression was significantly decreased following the treatment of MGC-803 cells with deguelin alone or deguelin-cisplatin in combination for 48 h, while no significant differences were identified in the expression of ERCC1 and XRCC1. However, treatment with cisplatin alone was not observed to alter the expression of BRCA1, ERCC1 or XRCC1 (Fig. 5).

## Discussion

Gastric cancer is prevalent in a number of countries worldwide, accounting for 7.8% of all novel cancer cases and ~800,000 mortalities per year (1,24). Despite the progress in recent years, the treatment outcome of gastric cancer remains unsatisfactory with a five-year survival rate of <30%. Therefore, novel drugs are urgently required to improve the treatment of this malignancy.

Cisplatin, a representative platinum-based drug, is one of the most commonly used drugs in the therapy of malignancies, such as gastric cancer (25,26). In cancer cells, platinum-based drugs efficiently bind to DNA to form a variety of covalent adducts, which block replication and transcription and eventually induce cell apoptosis or necrosis (27). As DNA damage exerts antitumor effects, increasing DNA repair may decrease the sensitivity of cells or induce resistance to platinum-based drugs (28,29). However, if DNA repair is inhibited, the platinum-based drugs may exhibit more efficient antitumor effects.

Deguelin, a naturally occurring rotenoid, is capable of inhibiting phosphatidylinositide 3-kinase in premalignant and malignant human bronchial epithelial cells (13). It has been reported that deguelin markedly enhances the sensitivity of human U937 leukemia cells and acute myeloid leukemia blasts to chemotherapeutic drugs via the downregulation of Akt phosphorylation (30). However, the influences of deguelin on platinum based drugs remain unclear. Therefore, in the present study, the interaction between deguelin and cisplatin was examined in the gastric cancer MGC-803 cell line.

In the present study, deguelin was found to inhibit the proliferation of gastric cancer cells in a time- and dose-dependent manner. Furthermore, DNA damage in cells was induced by deguelin via the downregulation of DNA repair genes, consistent with a previous report (16). When combined with deguelin, the antitumor effect of cisplatin was enhanced. Furthermore, the combination of deguelin with cisplatin was found to increase the therapeutic efficacy of each drug, resulting in a synergistic interaction (CI<1). In addition, these results indicated that the inhibition of DNA damage repair via the downregulation of BRCA1 underlies the synergistic effect of deguelin and cisplatin.

In conclusion, deguelin in combination with cisplatin exhibits a synergistic and significant antitumor effect in gastric cancer cells. This may have promising therapeutic value for gastric cancer and thus warrants further investigation.
